# Frailty and *Caenorhabditis elegans* as a Benchtop Animal Model for Screening Drugs Including Natural Herbs

**DOI:** 10.3389/fnut.2018.00111

**Published:** 2018-11-26

**Authors:** Katsuyoshi Matsunami

**Affiliations:** Department of Pharmacognosy, Graduate School of Biomedical and Health Sciences, Hiroshima University, Hiroshima, Japan

**Keywords:** frailty, *Caenorhabditis elegans*, drug screening, natural herbs, model animal

## Abstract

*Caenorhabditis elegans* has been used in research for years to clarify the genetic cascades and molecular mechanisms of aging, longevity, and health span. Health span is closely related to frailty; however, frailty has a different concept and is evaluated using various parameters in humans, such as Fried's Frailty Criteria. The *C. elegans* model has several advantages when performing a chemical screen to identify drug candidates. Several mouse models of frailty were recently developed, including a homozygous *IL-10* knockout. These mouse models are useful for understanding human frailty; however, they are not appropriate for primary drug screening because they require large spaces, expensive cost, and time consuming assessments. Therefore, a combination of these models may be a promising tool for discovering drugs and understanding the mechanisms of frailty. In addition, natural products, and herbs are attractive sources of novel drugs with pharmacological activity and low toxicity, in fact, over 60% of currently-available drugs are estimated to be related to natural compounds. In this review, the possibility of identifying natural agents (i.e., herb extracts and compounds) that could improve frailty are proposed, and the advantages and limitations of these models are also discussed.

## Introduction

Frailty is a complex geriatric syndrome that is associated with increased vulnerability and a reduced physiological reserve that could lead to adverse health outcomes such as an increased risk of falls, dependency, disability, hospitalization, and mortality (1, 2). Shimada et al. performed a population-based survey to ascertain the prevalence of frailty in 5104 older (≥65 years; mean age: 71 years) Japanese adults (3). The authors showed that the rate of frailty increased with age and the overall prevalence of frailty was 11.3% (5.6, 7.2, 16, and 34.9% in the 65–69, 70–74, 75–79, and ≥80 age groups, respectively) (3). The global prevalence of physical frailty assessed using Fried's criteria was summarized by Choi et al. (4). The authors included data from the USA, Europe, and Asia and found that the prevalence of frailty and prefrailty varied between 4.9 and 27.3%, and 34.6 and 50.9%, respectively (4).

The global population is aging rapidly. In 2015, ~8.5% of the global population (7.3 billion) was aged ≥65. The number of older individuals is continuing to increase and is estimated to reach 12.0% (equivalent to 1 billion people) of the global population by 2030, and 16.7% (9.4 billion) by 2050. This increase in population is mainly due to low fertility and increased life expectancy (5).

Frailty is associated with multisystem impairments and chronic disease risk factors including cognitive impairment, diabetes, osteoporosis, chronic cardiovascular disease, kidney disease, malnutrition, chronic inflammation, and sarcopenia (2, 6, 7). These risk factors are related to the quality of life of older people and eventual mortality (2, 8–10). Therefore, given the emergent trend in global aging, interventions against frailty are a major concern (11).

## Frailty criteria in humans

Clinical frailty criteria were first introduced in cardiovascular health studies and included unintentional weight loss, self-reported fatigue or feelings of unusual tiredness or weakness, low activity levels (based on the frequency and duration of physical activity), slow walking times, and low grip strength (based on body mass index). These criteria were used to define frailty as either non-frail, prefrail, or frail (Fried Frailty Index) (10). In addition, the Clinical Global Impression of Change in Physical Frailty (CGIC-PF) (12); the Fatigue, Resistance, Ambulation, Illnesses, and Loss of Weight questionnaire (FRAIL scale) (13); the Canadian Study of Health and Aging (CSHA) clinical frailty scale (14); and the Gerontopole Frailty Screening Tool (GFST) (15) are also used to assess frailty.

Frailty is considered to be a dynamic process of accelerated aging in the absence of disability (16); however, it is difficult to understand the molecular and genetic mechanisms of human aging and frailty due to the ethical problem, genetic diversity, and lifestyle variability of the older human population.

## Mammalian models of frailty

Several mouse models and their assessment tools were recently developed and provided an invaluable opportunity to conduct research into the mechanisms of frailty, the interventions to ameliorate frailty, and the effects of frailty on adverse outcomes using validated models (17–24).

Parks et al. were the first to attempt to establish a mouse frailty scale, which contained 31 parameters including activity levels, hemodynamics, body composition, and serum analysis. The authors found that frailer older mice showed the greatest myocyte hypertrophy and the worst peak contraction (17). However, this assessment had its limitations, as the experimental equipment used is uncommon for most laboratories. Whitehead et al. were the next to report an animal frailty index that contained visually-inspected and non-invasive assessment parameters (18). Liu and Graber et al. reported another mouse frailty index that used an activity wheel, a rotarod, and an inverted-cling grip device and resembled the Fried Frailty Test used in humans (19). These criteria provide a platform for validated preclinical animal models and have been implemented for a wide range of applications (25).

Graber et al. evaluated the effects of physical interventions in old mice using the mouse frailty index established by themselves (19). The authors found that voluntary aerobic exercise significantly improved the frailty score in C57BL/6 mouse (26). The effects of dietary and pharmaceutical interventions on frailty were also investigated using the criteria developed by Whitehead (18), and these treatments significantly reduced the mouse frailty index in DBA/2J and C57BL/6J mouse (27).

In addition, a rat frailty index was also recently developed (28, 29). Miller et al. selected criterion tests and configured appropriate cutoff points and indices to identify frailty in aged Fischer F344 rats. This model adapted existing clinical and preclinical indices, including grip strength, endurance, walking speed, and physical activity, that were used in human and mouse frailty indices. Yorke et al. also independently developed a rat frailty index for aged Fischer F344 rats using 27 criteria (29).

Animal models, such as transgenic and gene knockout mice, continue to be useful tools for preclinical studies in various diseases. Walston et al. reported a frail mouse model (i.e., IL-10^tm/tm^) and characterized the physical and biological features to be similar to those seen in human frailty (30, 31). Mice carrying a homozygous targeted mutation of the *IL-10* gene (IL-10^tm/tm^) were first generated by Kuhn et al. (32). This mouse was developed as a model of colitis but was found to exhibit a frail phenotype that was characterized by inflammation, reduced muscle strength, and a reduced health span. Aged IL-10^tm/tm^ mice showed stiffer vasculature, which was in accordance with the increased COX-2 activity and thromboxane A2 receptor activation (33). In addition, ATP synthesis and the free energy released from ATP hydrolysis in skeletal muscle was reduced in this frail mouse model, which provides some mechanistic insight into skeletal muscle weakness in mouse and human frailty (33). Higher glucose level may be a risk factor for frailty in older human adults (34), and frail and prefrail older adults present lower estimated resting metabolic rate (eRMR) than non-frail adults, together with lower expired volume (Ve) and oxygen consumption (VO_2_) values that were partially compensated by an respiratory frequency (RF) increase (35).

Westbrook et al. investigated the older IL-10^tm/tm^ mice concerning on metabolic parameters shown in older humans, i.e., glucose metabolism, oxygen consumption (VO_2_), respiratory quotient (RQ), spontaneous locomotor activity, body composition, and plasma adipokine levels. Interestingly, VO_2_, fat mass, plasma adiponectin, and leptin were decreased with age in IL 10 ^tm/tm^ mice compared to controls, although insulin sensitivity, glucose homeostasis, locomotor activity, and RQ were not significantly altered. These findings suggest that frailty of this mouse model may be caused by reduction of fat mass, hormonal secretion and energy metabolism (36). Deepa et al. reported a new mouse model of frailty, Sod1KO mouse lacking the antioxidant enzyme Cu/Zn superoxide dismutase (24). The Sod1KO mice exhibited some features of human frailty including weight loss, weakness, low physical activity levels, exhaustion, increased inflammation, and sarcopenia. Dietary restriction in the Sod1KO mouse prevented the progression of frailty (24).

Thus, mouse frailty indices and normal and genetically-modified mouse models are important research tools that allow us to understand the biological mechanisms of frailty and evaluate novel interventions to ameliorate frailty. However, drug screening in mammalian models is expensive, time-consuming, requires large amount of drug candidates, and is relatively low throughput for many laboratories, although mammalian models are the most reliable and important platforms for preclinical studies.

## *Caenorhabditis elegans*: a benchtop animal model for initial drug screening

Non-mammalian model organisms are attractive options for discovering antifrailty drugs. Among the various well-known model organisms (i.e., *Danio rerio* (zebrafish), *Drosophila melanogaster* (fruit fly), and the nematode, *C. elegans*), *C. elegans* is the most studied animal in the field of aging, lifespan, and health span (37). It was first introduced to the field of basic biology in 1963 and has been used in a variety of studies assessing development (38), cell death (39), RNA interference (RNAi) (40), and aging (41). In fact, the genetic basis of aging has first recognized in *C. elegans* via the discovery of *age-1, daf-2*, and *daf-16* mutants (42–44). The lifespan was doubled by mutations in the *age-1* (PI3K, phosphoinositide 3-kinase) or *daf-2* (InR, insulin/IGF-1 receptor) genes, and reduced in the *daf-16* (FOXO transcription factor) mutant. Following this pioneering discovery, many researchers have used this model to focus on the genetic analysis and exploration of chemical interventions for longevity.

**Figure 1:**
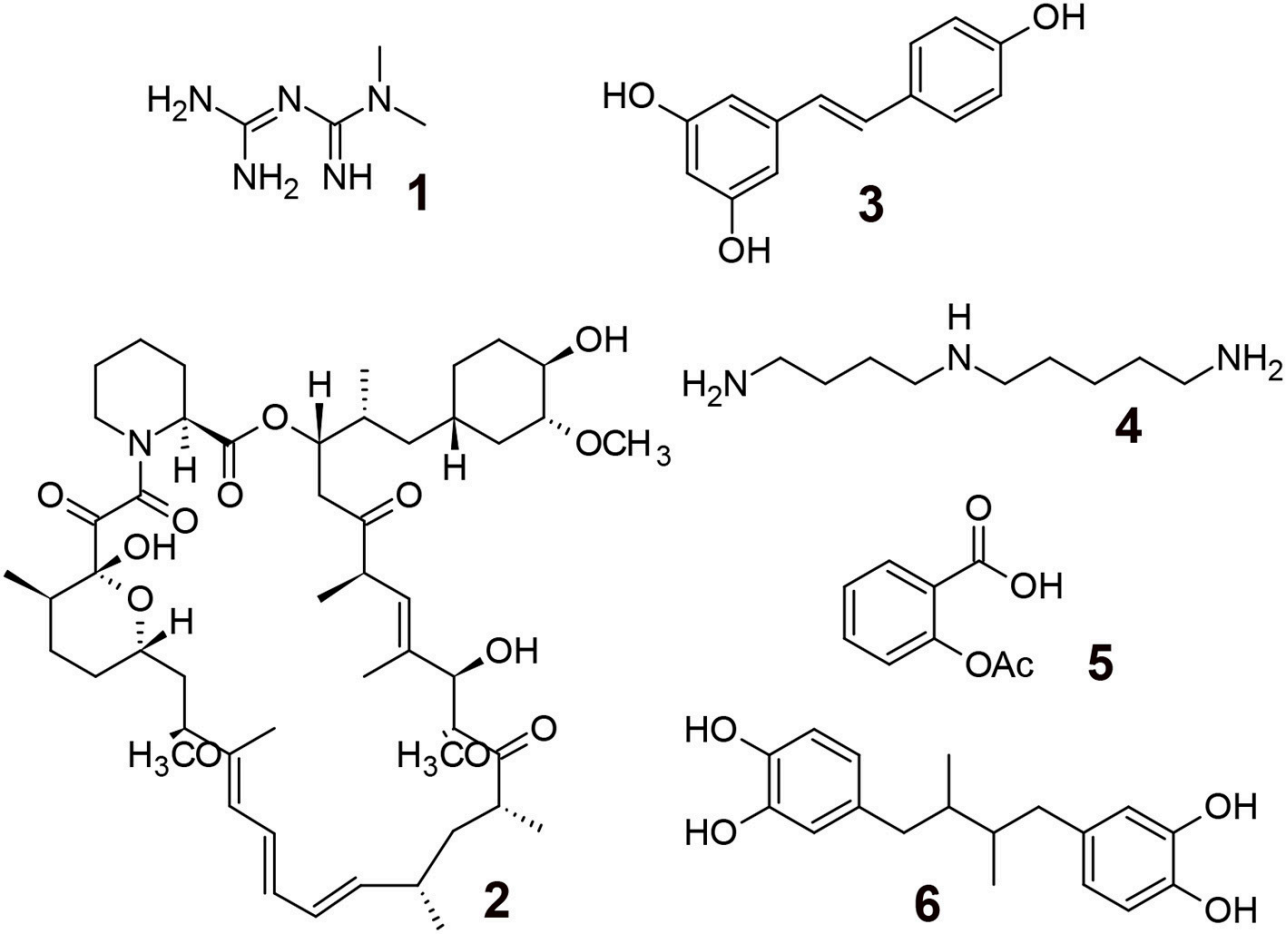
Aging modulating compounds. 1; Metformin (biguanide antiglycemic agent, AMPK activation), 2; Rapamycin (immune suppressing agent, mTOR inhibitor), 3; Resveratrol (polyphenol, sirtuin activator), 4; Spermidine (polyamine, induction of autophagy), 5; Aspirin (COX inhibitor, antithrombosis, antioxidant), 6; Masoprocol (catechol, antioxidant, antiinflammation).

The pioneering research using *C. elegans* revealed that numerous pathways, including insulin/insulin-like growth factor-1 signaling, target of rapamycin signaling, AMP-activated protein kinase, sirtuins, mitochondrial stress-signaling pathways, and caloric restriction (45), were conserved in different organisms (e.g., *C. elegans, D. melanogaster*, and *Mus musculus*), and several chemicals have been investigated as potential candidates for extending life-span (41, 46, 47) (Figure 1).

*C. elegans* have many desirable features for drug discovery, such as their ease of maintenance in the laboratory, their transparent bodies for anatomical observation, their high genetic homology (60–80%) to humans, the publication of the complete genome sequence (48–50), conserved biological molecular responses, essentially no ethical problems, their high fertility rates (~250 eggs/worm within several days), and the availability of molecular biology tools (i.e., transgenic, gene knockouts, and RNAi knockdowns) (37). In addition, their short lifespan (~3 weeks) and small size are favorable for the screening of antiaging drugs due to the reduced experimental costs and their capacity for high throughput (51) (Table 1).

**Table 1:** Feature of *C. elegans*.

•Multicellular animal
•Small size (~1 mm in length)
•Short life cycle (~3 days)
•High progeny production (~250 offspring in ~3 days)
•Conservation of cellular processes and genes
(Homologs have been identified for 60–80% of human genes)
•ADMET characteristics
•Low husbandry and animal costs
•Simple and high-throughput screening assays
•Availability of mutant and transgenic strains

Frailty is defined as a condition of decreased physiological reserves by multisystem dysregulation and increased vulnerability to stressors (2). Similarly, aging is defined as the decreased adaptability to internal and external stress and increased vulnerability to disease and mortality by an accumulation of deficits derived from the progressive structural and functional decline of proteins, cellular organelles, tissues, and organs (52, 53). Both of these definitions have a lot in common, although the phrases are different.

Moreover, many age-associated features described in mammals, including neuromuscular degeneration, weakness to stressors, elevated infection levels, decreased physiological activity, and increased mortality, are also observed in *C. elegans* models (54).

Aging in *C. elegans* is also characterized by a severe loss of muscle mass and function (sarcopenia) (55), which gradually interferes with movement and the ingestion of food. Muscle mitochondrial energy dysregulation (56–58) and an accumulation of oxidative damage and aggregates in muscle cells are also likely to be related to muscle dysfunction in aged *C. elegans* (59, 60).

Several research papers have recently documented the relationship between lifespan, health span, and frailty in *C. elegans*. Newell et al. reported that mutants of age-related pathway genes in *C. elegans* showed that long-lived mutants displayed prolonged midlife movement and did not prolong the frailty period assessed by locomotor decline (56, 61); however, Bansal et al. previously reported controversial results showing that some long-lived mutants increased the proportion of the frailty period rather than health span (62).

When considering an improvement in quality of life, the health span-to-gerospan ratio is much more important than lifespan extension alone (62). Therefore, interventions focusing on the health span along with lifespan of the aging population are favorable.

Aging is characterized by muscular dysfunction as observed in sarcopenia and frailty. These two phenotypes are substantially overlapped with each other, and many of the adverse outcomes of frailty are probably mediated by sarcopenia (63–65).

In aged *C. elegans*, a gross decline in general behaviors (i.e., locomotion and feeding) is correlated with degeneration of muscle structure and contractile function (55). Loss of muscle mass is the major cause of aging-related functional decline, sarcopenia, and frailty. Several factors are correlated with sarcopenia including contraction-related cellular injury, oxidative stress, endocrine changes, and a reduced regenerative potential. In addition, both functional and structural decline in the pharynx during aging is significantly delayed in mutants with reduced muscle contraction rates that affect the initiation and progression of sarcopenia during aging (55, 60).

In addition, *C. elegans* containing a transgenic strain of human amyloid beta 1–42 (Aβ) under a neuron-specific promoter, as an Alzheimer's disease model, showed eight-fold slower locomotion than wildtype worms. This model seems consistent with the frailty seen in Alzheimer's patients (66–68). Tan et al. found a high prevalence of frailty in Parkinson's disease recently (69). The transgenic *C. elegans* of human α-synuclein gene as a Parkinson's disease model has been used for the demonstration of a natural product, squalamine, for the reduction of α-synuclein aggregation and muscle paralysis (70).

Sonowal et al. recently showed that small molecules, indole and derivatives, e.g., indole-3-carboxaldehyde and indole acetic acid, from commensal microbiota could extend the health span (i.e., the non-frailty period) of *C. elegans*. These compounds were also effective in *D. melanogaster* and *M. musculus*, therefore these compounds may become potential drug candidates to extend the health span and reduce frailty in humans (71). In this research, a lifespan assay (to measure longevity), two locomotion assays related to sarcopenia (e.g., a thrashing motility assay and a pharyngeal pumping assay), and a heat-stress assay (to measure vulnerability) were performed in *C. elegans*. These assays are popular, reliable, and well-studied so far as the *C. elegans* health span assay (72).

## Conclusion

According to the 2016 review by Newman and Cragg, natural products continue to be an important source of clinical trial drugs and drug candidates; for example, ~65% of small-molecule drugs approved from 1981 to 2014 were directly or indirectly related to natural compounds (73). Among the various natural resources (i.e., plants, microbials, and marine organisms), plants have a long history of medicinal use that goes back to the ancient records of Mesopotamia, which chronicled their use in the treatment of various diseases. The total number of higher plants species in the world is estimated to be around 250,000; however, many of these remain to be characterized phytochemically. Thus, natural products and herbs are still attractive sources of novel drugs with pharmacological activity and low toxicity (74).

The *C. elegans* model is advantageous when performing a chemical screen to identify drug candidates to increase the health span. Among the various health span assays, longevity, thrashing motility, pharyngeal pumping, and heat stress assays are preferable as they have already been successfully utilized for the discovery of candidate compounds (71).

**Scheme 1:**
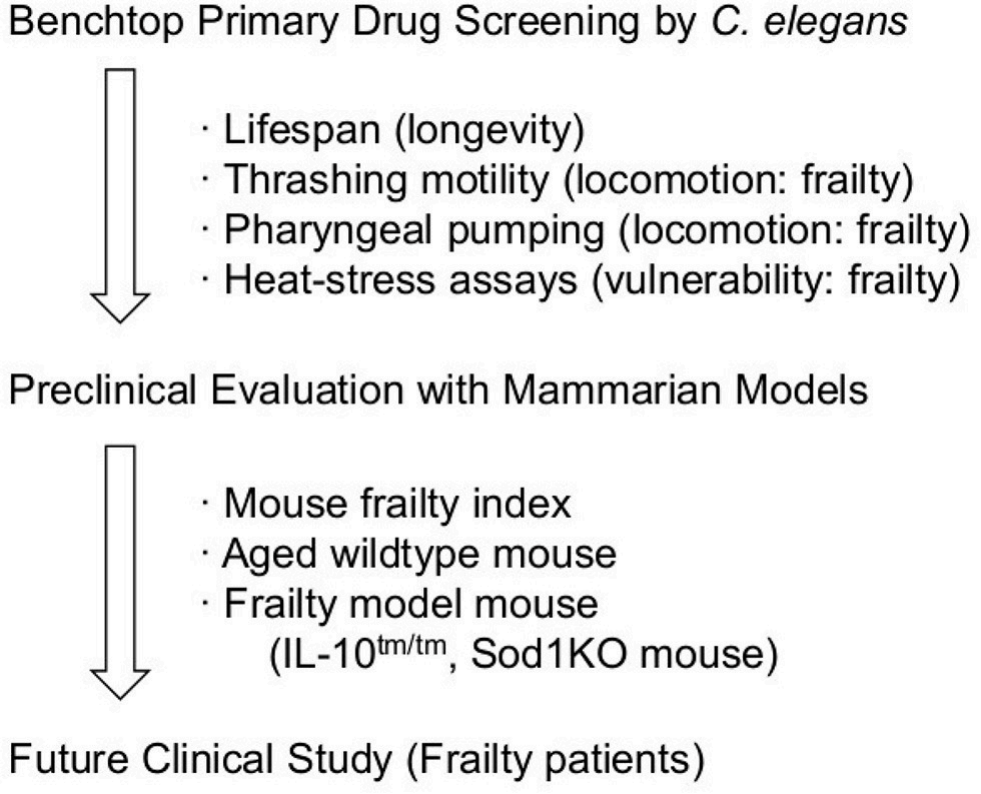
Plausible work flow for anti-frailty agents.

Wildtype and genetically-modified mouse models are useful for estimating efficacy on human frailty; however, they have several disadvantages for primary drug screening because of their scale, cost, and labor intensiveness. Therefore, the combination of these models may provide a promising workflow to discover drugs and understand the mechanism of frailty (Scheme 1).

## Author contributions

The author confirms being the sole contributor of this work and has approved it for publication.

### Conflict of interest statement

The author declares that the research was conducted in the absence of any commercial or financial relationships that could be construed as a potential conflict of interest. The handling Editor declared a shared affiliation, though no other collaboration, with the author KM.
